# Extracting disease risk profiles from expression data for linkage analysis: application to prostate cancer

**DOI:** 10.1186/1753-6561-1-s1-s82

**Published:** 2007-12-18

**Authors:** G Bryce Christensen, Lisa A Cannon-Albright, Alun Thomas, Nicola J Camp

**Affiliations:** 1 Department of Biomedical Informatics, University of Utah, 391 Chipeta Way Suite D, Salt Lake City, Utah 84108-1266, USA

## Abstract

The genetic factors underlying many complex traits are not well understood. The Genetic Analysis Workshop 15 Problem 1 data present the opportunity to explore whether gene expression data from microarrays can be utilized to define useful phenotypes for linkage analysis in complex diseases. We utilize expression profiles for multiple genes that have been associated with a disease to develop a composite 'risk profile' that can be used to map other loci involved in the same disease process. Using prostate cancer as our disease of interest, we identified 26 genes whose expression levels had previously been associated with prostate cancer and defined three phenotypes: high, neutral, or low risk profiles, based on individual expression levels. Linkage analyses using MCLINK, a Markov-chain Monte Carlo method, and MERLIN were performed for all three phenotypes. Both methods were in very close agreement. Genome-wide suggestive linkage evidence was observed on chromosomes 6 and 4. It was interesting to note that the linkage signals did not appear to be strongly influenced by the location of the original 26 genes used in the phenotype definition, indicating that composite measures may have potential to locate additional genes in the same process. In this example, however, extreme caution is necessary in any extrapolation of the identified loci to prostate cancer due to the lack of data regarding the behavior of these genes' expression level in lymphoblastoid cells. Our results do indicate there exists potential to augment our current knowledge about the relationships among genes associated with complex diseases using expression data.

## Background

Recent advances in biotechnology have resulted in an explosion of genotypic and phenotypic data. Millions of single-nucleotide polymorphisms (SNPs) can quickly and accurately be genotyped, and microarray technology has made it possible to simultaneously assess the expression levels for many thousands of genes. The question becomes: what knowledge can we extract from these extensive data sources with respect to disease susceptibility? And how? The Genetic Analysis Workshop 15 (GAW15) Problem 1 data presents a unique opportunity to explore whether gene expression data from microarrays can be used to define useful phenotypes for linkage analysis to better understand disease susceptibility. The expression data provided for Problem 1 includes 3554 genes that were previously established to have greater variation between individuals than within individuals. These expression levels are reasonable candidates for use as phenotypes in linkage analysis [1].

For the majority of complex traits the underlying genetic factors are not fully understood, but for many, certain genes and/or genetic pathways have been implicated or related to the trait through expression experiments. The expression levels of a gene may be controlled by regulatory genes elsewhere in the genome, and the expression of multiple genes can be regulated by a common transcription factor [2]. Hence, linkage analysis of gene expression levels could conceivably identify regulatory loci associated with that gene. Further, and more related to a disease end-point, if several genes are known to be related to a given trait, it is also conceivable that their expression levels could be combined to create a phenotype to be used in linkage analysis to identify loci that are involved in disease susceptibility, perhaps through membership in the pathway or interaction (epistasis) with the known genes.

In this study we explore whether gene expression profiles for genes that have been associated with a disease can be used to map other genes that are involved in the disease process or highlight genes within the pathways that are key factors. Here we specifically examine the approach for prostate cancer.

Research has consistently shown that genetics plays a critical role in prostate cancer development, but the identification of specific genes has proven to be very difficult. Hereditary prostate cancer is a complex disease involving numerous genes and variable phenotypic expression [3]. Recent research has demonstrated great potential for the use of proteomic profiling and other biomarkers for prostate cancer diagnostics [4]. One such study was able to discriminate between benign and cancerous prostates with perfect sensitivity in men with elevated prostate specific antigen (PSA) levels using serum proteomic profiling [5]. The GAW15 Problem 1 data provide an opportunity to explore whether gene expression levels from lymphoblastoid cells can be used to develop a prostate cancer profile phenotype for use in linkage analysis. Using expression data from 26 genes whose expression levels had previously been reported to be associated with prostate cancer [6], we defined individuals as having high, neutral, or low risk profiles based on their individual expression levels. Here we present the results of linkage analyses based on those phenotypes.

## Methods

**Figure 1:**
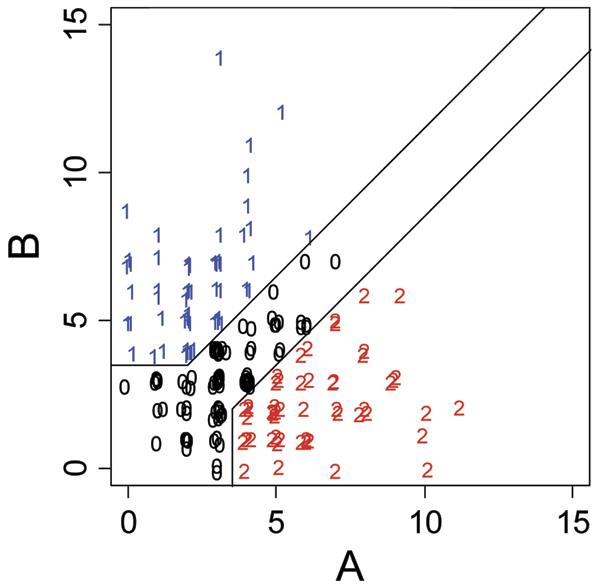
**Phenotype distribution**. A, the number of genes expressed more than 1 SD in the direction associated with prostate cancer for an individual; B, the number of genes expressed greater than 1 SD in the opposite direction; 0, neutral risk status; 1, low-risk profile; 2, high-risk profile.

Ashida et al. identified 21 genes that are commonly up-regulated and 63 genes that are commonly down-regulated in the transition from normal epithelium to cancerous and/or prostatic intraepithelial neoplasia (PIN) [6]. Of these 84 genes, 26 were included in the data for Problem 1. These 26 genes are listed in Table 1. Using the expression data for the 194 individuals in the Problem 1 data, we scaled the expression levels for each of these 26 genes to fit a standard normal distribution with mean of 0 and variance of 1. Two statistics, ***A ***and ***B***, were then computed for each individual. ***A ***represented the number of genes for which the expression levels was greater than 1 standard deviation in the direction associated with prostate cancer. ***B ***represented the number of genes for which the expression level was greater than 1 standard deviation in the opposite direction. One standard deviation was selected arbitrarily as a threshold to ensure that the expression values were distant from the center of the distribution, while allowing for a sufficient number of informative subjects in the subsequent linkage analysis. An individual was considered to be in the "high-risk profile" group if ***A ***≥ 4 and ***A***-***B ***≥ 2. Individuals were classified to be in the "low-risk profile" group if ***B ***≥ 4 and ***B***-***A ***≥ 2. All other subjects were classified as "neutral" and were considered as "unknown" in all linkage analyses. This classification system was devised to distribute the influence of the 26 genes on the assigned risk profiles and to prevent outlying expression levels of individual genes from having undue influence. As shown in Figure 1, 53 subjects (25 male and 28 female) were classified with high-risk profiles, 57 (32 male and 25 female) with low-risk profiles, and 84 (42 male and 42 female) as neutral (unknown). While women are not susceptible to prostate cancer, they may still carry the susceptibility genes, hence in our analyses both males and females are included. Figure 1 shows a scatter plot of the values of ***A ***and ***B ***for each individual and the categorization to the high-risk, low-risk, and neutral groups.

**Table 1:** Genes used to create phenotype definition

Gene	Location
Up-regulated	
*ABCC4*	chr13q32
*AMACR*	chr5p13.2-q11.1
*MIPEP*	chr13q12
*PRC1*	chr15q26.1
*SMS*	chrXp22.1
Down-regulated	
*ANXA2*	chr15q21-q22
*ARHGDIB*	chr12p12.3
*ASS*	chr9q34.1
*BHLHB2*	chr3p26
*CD74*	chr5q32
*CSPG2*	chr5q14.3
*CUTL1*	chr7q22.1
*CX3CL1*	chr16q13
*FHL2*	chr2q12-q14
*FLNA*	chrXq28
*GATA3*	chr10p15
*GBP2*	chr1p22.2 *I*
*ER3*	chr6p21.3
*IRF1*	chr5q31.1
*KRT7*	chr12q12-q13
*LY6E*	chr8q24.3
*MMP7*	chr11q21-q22
*MYL9*	chr20q11.23
*SERPINB1*	chr6p25
*TOP2B*	chr3p24
*WFDC2*	chr20q12-q13.2

Three phenotype models were considered. The first model (*"FULL"*) included the high-risk profile individuals as "affected" and the low-risk profile individuals as "unaffected"; neutrals were "unknown". The second model (*"HIGH"*) included the high-risk profile individuals as "affected" and all others as "unknown". The third model (*"LOW"*) included the low-risk profile individuals as "affected" and all others as "unknown". This final phenotype model is akin to an analysis searching for protective genes. For the *FULL *and *HIGH *phenotype models, 10 of the 14 CEPH (Centre d'Etude du Polymorphisme Humain) pedigrees were informative for linkage, with between two and eight affected subjects per pedigree. Thirteen pedigrees were informative in the *LOW *analysis, with up to nine affected subjects.

Dominant and recessive parametric linkage analyses were performed using MCLINK, which uses Markov-chain Monte Carlo simulation methods to sample haplotype configurations to estimate the LOD statistic [7]. The inheritance model for the analysis was based on the "Smith" model used to map the *HPC1 *locus, but without the specificity to males [8], and assumes a population prevalence of 0.003 for the mutant allele. Genotypes for a genome-wide panel of 2882 SNP markers were provided by GAW15. The genetic map used in the analysis was based on the Rutgers genetic map, with the positions of SNPs for which genetic map position was not available interpolated from flanking markers based on physical location [9]. Any SNP located less than 0.001 cM from the preceding SNP was eliminated from the initial analysis. After completing the initial analyses, the best linkage peaks were identified and those regions were reanalyzed using a reduced marker map, with a minimum spacing of 0.3 cM between SNPs [10]. This was done to control for the possible effects of linkage disequilibrium (LD), which may inflate LOD scores. The linkage statistics for these chromosomes were then confirmed by performing both parametric and model-free analyses with MERLIN [11]. Linked pedigrees (LOD > 0.588, which represents a nominal, uncorrected *p *< 0.05 for an individual pedigree) were identified in the regions with HLOD > 1.9 (genome-wide suggestive evidence for linkage [12]) and gene expression profiles within those pedigrees were inspected to ensure that the linkage evidence was not correlated with the expression levels of any specific genes.

## Results

**Figure 2:**
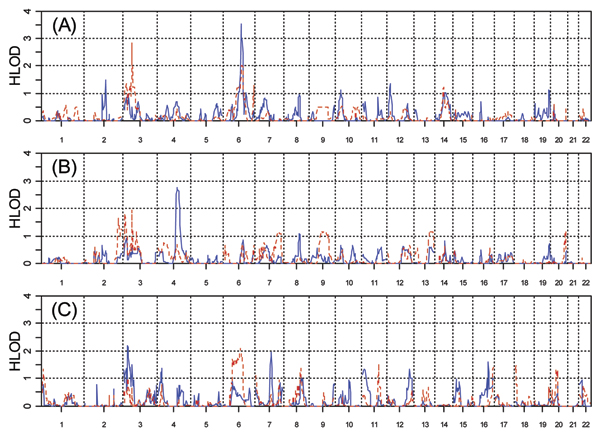
**Genome-wide HLOD statistics**. A, FULL analysis model; B, HIGH model; C, LOW (protective) model. The solid line represents the dominant inheritance model and the broken line represents the recessive model in each figure. HLOD values are shown on the vertical axis, and chromosome number is shown on the horizontal axis.

The genome-wide scan results showing the HLOD statistic for all models are shown in Figure 2. Significant linkage evidence was observed on chromosome 6q (HLOD = 3.51). Other peaks over HLOD = 1.9 were observed on chromosomes 3, 4, and 7. Only the peaks on chromosomes 4 and 6 retained at least suggestive linkage evidence with the reduced marker set without LD.

**Figure 3:**
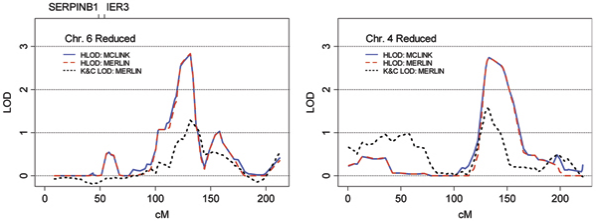
**Analyses with increased marker spacing**. Detail of chromosome 6 from the *FULL *phenotype model and chromosome 4 from the *HIGH *phenotype model using a minimum marker spacing of 0.3 cM. The solid blue line in each panel represents the dominant HLOD statistic as calculated by MCLINK, the broken red line shows the dominant HLOD from Merlin, and the black dotted line shows the model-free Kong and Cox LOD score from MERLIN. The locations of genes included in the phenotype definition are indicated at the top of each frame.

The strongest linkage signal observed in the *FULL *analysis, and the best result overall, was HLOD = 3.51 at marker rs1491074 under the dominant model on chromosome 6q. As is shown in Figure 3, 2 of the 26 genes used in creating the phenotype (*SERPINB1 *and *IER3*) are located on chromosome 6, however, they are not situated near the linkage peak. Chromosome 6 was reanalyzed using a map with increased marker spacing (which reduced the number of SNPs used from 101 to 70 and excluded SNP rs1491074) and the maximum HLOD fell to 2.82, suggesting the possible influence of LD in the initial result. This result was confirmed using MERLIN. The model-based HLOD statistic from MERLIN was very similar to results from MCLINK for both the full and reduced marker sets, although the model-free Kong and Cox LOD score did not perform well.

The best result in the *HIGH *analysis was HLOD = 2.75 at marker rs885103 under the dominant model on chromosome 4q. Three pedigrees were linked to the locus with individual LOD scores > 0.588. None of the genes used to determine the phenotype were located on chromosome 4. Linkage results were unchanged when the peak was reanalyzed with the reduced marker map, as shown in Figure 3. MERLIN analysis confirmed the parametric linkage result from MCLINK.

## Discussion

One concern of a study based on expression levels of known genes is that a linkage analysis may simply map back to the genes used to construct the phenotype. This did not appear to be the case for this study. None of the genes were located near our best results on chromosomes 6 and 4. Our phenotype definition was simplistic, but was designed to limit the influence of individual genes on the phenotype, and thereby enhance the likelihood of identifying a locus related to the entire set. It is interesting to note that the regions we identified on chromosomes 6 [13,14] and 4q [15,16] have each been implicated in previous linkage analyses for prostate cancer. However, it is premature to consider these as replications, because without data indicating that the expression levels seen in tumors [6] are also representative in lymphoblastoid cells, there is no evidence that the risk profiles we created are actually related to prostate cancer. This is a major weakness of our particular example, and perhaps illustrates the weakness of such approaches in general-that is, much of the experimental data is still missing and will be expensive to generate.

Because the true locations of any genes that interact with or modify the 26 we studied are not known, the statistical power of this approach can not be properly evaluated. However, with the 14 CEPH pedigrees, we were able to generate linkage peaks that appeared distinct from background noise. Further, we know that the linkage evidence observed was not influenced by the linkage analysis method chosen, as both MCLINK and MERLIN produced almost identical results. Recognizing the limitations of the data available, we present these results as proof of concept that the expression levels of several related genes can be combined to create a phenotype that can reasonably be used in linkage analysis. Such an approach could identify loci that regulate or contribute to disease pathways. More work is needed to refine and test the methodology, and more experimental data is needed to correlate tissue and lymphoblastoid expression levels, but the approach appears to have the potential to augment our current knowledge about the genetic basis of complex diseases.

## Competing interests

The author(s) declare that they have no competing interests.
